# Temporal Trends in the Characteristics of Children at Antiretroviral Therapy Initiation in Southern Africa: The IeDEA-SA Collaboration

**DOI:** 10.1371/journal.pone.0081037

**Published:** 2013-12-09

**Authors:** Mary-Ann Davies, Sam Phiri, Robin Wood, Maureen Wellington, Vivian Cox, Carolyn Bolton-Moore, Venessa Timmerman, Harry Moultrie, James Ndirangu, Helena Rabie, Karl Technau, Janet Giddy, Nicola Maxwell, Andrew Boulle, Olivia Keiser, Matthias Egger, Brian Eley

**Affiliations:** 1 School of Public Health and Family Medicine, University of Cape Town, Cape Town, South Africa; 2 Lighthouse Trust Clinic, Kamuzu Central Hospital, Lilongwe, Malawi; 3 Gugulethu Community Health Centre and Desmond Tutu HIV Centre, Institute of Infectious Diseases and Molecular Medicine, University of Cape Town, Cape Town, South Africa; 4 Newlands Clinic, Harare, Zimbabwe; 5 Médecins Sans Frontières South Africa and Khayelitsha ART Programme, Khayelitsha, Cape Town, South Africa; 6 Centre for Infectious Disease Research in Zambia, Lusaka, Zambia and University of North Carolina at Chapel Hill, Chapel Hill, North Carolina, United States of America; 7 Knowledge Translation Unit, University of Cape Town Lung Institute, Cape Town, South Africa; 8 Wits Reproductive Health and HIV Institute, Harriet Shezi Children's Clinic, Chris Hani Baragwanath Hospital, Faculty of Health Sciences, University of Witwatersrand, Soweto, Johannesburg, South Africa; 9 Africa Centre for Health and Population Studies, University of KwaZulu-Natal, Somkhele, South Africa; 10 Tygerberg Academic Hospital, University of Stellenbosch, Stellenbosch, South Africa; 11 Empilweni Services and Research Unit, Rahima Moosa Mother and Child Hospital and University of Witwatersrand, Johannesburg, South Africa; 12 Sinikithemba Clinic, McCord Hospital, Durban, South Africa; 13 Institute of Social and Preventive Medicine (ISPM), University of Bern, Bern, Switzerland; 14 Red Cross Children's Hospital and School of Child and Adolescent Health, University of Cape Town, Cape Town, South Africa; UNAIDS, Trinidad and Tobago

## Abstract

**Background:**

Since 2005, increasing numbers of children have started antiretroviral therapy (ART) in sub-Saharan Africa and, in recent years, WHO and country treatment guidelines have recommended ART initiation for all infants and very young children, and at higher CD4 thresholds for older children. We examined temporal changes in patient and regimen characteristics at ART start using data from 12 cohorts in 4 countries participating in the IeDEA-SA collaboration.

**Methodology/Principal Findings:**

Data from 30,300 ART-naïve children aged <16 years at ART initiation who started therapy between 2005 and 2010 were analysed. We examined changes in median values for continuous variables using the Cuzick's test for trend over time. We also examined changes in the proportions of patients with particular disease severity characteristics (expressed as a binary variable e.g. WHO Stage III/IV vs I/II) using logistic regression. Between 2005 and 2010 the number of children starting ART each year increased and median age declined from 63 months (2006) to 56 months (2010). Both the proportion of children <1 year and ≥10 years of age increased from 12 to 19% and 18 to 22% respectively. Children had less severe disease at ART initiation in later years with significant declines in the percentage with severe immunosuppression (81 to 63%), WHO Stage III/IV disease (75 to 62%), severe anemia (12 to 7%) and weight-for-age z-score<−3 (31 to 28%). Similar results were seen when restricting to infants with significant declines in the proportion with severe immunodeficiency (98 to 82%) and Stage III/IV disease (81 to 63%). First-line regimen use followed country guidelines.

**Conclusions/Significance:**

Between 2005 and 2010 increasing numbers of children have initiated ART with a decline in disease severity at start of therapy. However, even in 2010, a substantial number of infants and children started ART with advanced disease. These results highlight the importance of efforts to improve access to HIV diagnostic testing and ART in children.

## Introduction

The estimated number of children receiving antiretroviral therapy (ART) in southern and eastern Africa has increased several-fold from <50,000 in 2005 to 337,200 by the end of December 2010, corresponding to a coverage of 26% of those eligible for ART [1]. In addition, WHO 2010 guidelines recommended immediate ART for HIV infected children <2 years of age irrespective of disease severity, and ART at much higher CD4 thresholds than before in older children [2]. This means that more children are starting therapy and increasingly those initiating treatment should have less advanced disease. Furthermore, although drug development, availability and accessibility for children lags behind that of adults [3], [4], there have been moves towards less toxic and more effective first-line ART [2].

Timely ART initiation is important especially in infants and young children where disease progression is rapid and mortality is high [5], [6]. Even after ART initiation, early mortality remains very high in children with advanced disease, whereas starting ART with less advanced disease is associated with a good prognosis in terms of mortality, immunological, growth and neurodevelopmental outcomes [7], [8], [9], [10], [11], [12]. There have been few articles published examining temporal trends in children initiating ART in sub-Saharan Africa [13], [14], [15], [16]. During the early years of pediatric ART availability in South Africa between 2003 and 2007, ART initiation at 7 pediatric sites increased threefold and median baseline CD4% among children <5 years of age increased [17]. A subsequent South African study (2005–2009) found that in later years children had less severe disease and there were increasing proportions both of very young (<18 months) and older (≥10 years) children initiating therapy [13]. Comparison of secular trends in a study of 565 children initiating ART at urban and rural clinics in Zambia showed trends towards decreasing age and increasing CD4% with later years, however the study was small and changes were not all significant [14].

The International epidemiologic Databases to Evaluate AIDS Southern Africa (IeDEA-SA) collaboration now includes individualized data on >35,000 children ever started on ART across 12 sites in southern Africa. This provides an opportunity to examine temporal trends in baseline characteristics and regimen use in a very large cohort across a range of settings in 4 countries (Malawi, South Africa, Zambia and Zimbabwe). This is important to determine the extent to which HIV care and treatment programmes have expanded and timely ART initiation has improved in recent years, especially in relation to WHO and country guideline changes. It is also useful for planning programs. In addition to examining characteristics across the cohort as a whole, we particularly aimed to focus on changes in infants starting therapy as the Children with HIV Early Antiretroviral Therapy (CHER) trial has shown that ART initiation in infants before disease progression substantially reduces mortality and morbidity [2], [12], [18], [19].

## Methods

The IeDEA Southern Africa Collaboration has been approved by the University of Cape Town and University of Bern human research ethics committees. The requirement for informed consent has been waived as only anonymized data that is already collected as part of routine monitoring is contributed to the collaborative dataset. All sites have local institutional ethics approval to contribute data to IeDEA-SA analyses as follows: University of Zambia Biomedical Research Ethics Committee (Zambian Ministry of Health [MOH] – Centre for Infectious Disease Research in Zambia [CIDRZ]); University of Cape Town (Free State Province, Gugulethu Community Health Centre, Khayelitsha ART Programme, McCord Hospital, Red Cross Children's Hospital); University of KwaZulu Natal (Hlabisa HIV Care and Treatment Programme); University of Witwatersrand (Harriet Shezi Clinic, Rahima Moosa Mother and Child Hospital); Malawi National Health Sciences Research Ethics Committee (Lighthouse Clinic); Medical Research Council of Zimbabwe (Newlands Clinic) and University of Stellenbosch (Tygerberg Academic Hospital). The IeDEA-SA collaboration has been described previously and currently includes 12 HIV care and treatment programs for children in 4 southern African countries (www.iedea-sa.org) [9], [20]. Longitudinal data are collected prospectively at each site at ART initiation (baseline) and follow-up visits, using standardized definitions. Anonymized data are transferred to data centers at the Universities of Cape Town, South Africa, or Bern, Switzerland, in a standardized format and merged at regular intervals. We included all ART-naïve children (except for exposure to antiretrovirals to prevent mother to child transmission [PMTCT]) starting therapy at age <16 years between 1 January 2005 and 31 December 2010. Children starting before 2005 were excluded to limit the analysis to children likely to have presented after ART roll-out began. Children starting after 2010 were excluded as most cohorts transferred data during 2011 and so full data for that year is not available. The Hlabisa (South Africa) and Newlands (Zimbabwe) cohorts transferred data during 2010, and so did not contribute a full year of data for 2010. Children with a documented HIV-RNA measurement <400 copies/ml at baseline were excluded as they were unlikely to be ART-naïve.

Laboratory and anthropometric disease severity characteristics at ART start were the characteristics measured at the date closest to ART initiation within a window of −6 months to +7 days for CD4%/count, HIV-RNA and hemoglobin and a window of −1 month to +2 weeks for weight, height and body mass index (BMI). Weight-for-age, height-for-age and BMI-for-age z-scores (WAZ, HAZ, BAZ) were calculated using WHO 2006 standards [21]. BAZ was used rather than weight-for-height z-scores as these can be calculated for all ages, whereas weight-for-height standards are only available for children <5 years of age. Severe immune suppression was defined using the worst of CD4% and count, or whichever measure was available, using age-specific thresholds according to WHO guidelines [22]. Severe anemia was defined according to both hemoglobin and age using Division of AIDS criteria for grading of adverse events [23].

We examined changes over the duration of the program in continuous variables (using medians and interquartile ranges [IQR]) and in categorical variables (using proportions) for all children, and separately for those <1 year old at ART start. Changes in median values were assessed using the Cuzick's test for trend over the ordered groups of program year. Changes in the proportion of patients with a particular disease severity characteristic (expressed as a binary variable e.g. WHO Stage III/IV vs I/II) over the duration of the program were tested using unadjusted logistic regression with the disease severity characteristic as the dependent variable and examining the linear trend over program years as the independent variable. All analyses were done using STATA 12.0 (College Station, Texas, USA).

## Results

### Characteristics of all children starting therapy

**Figure 1:**
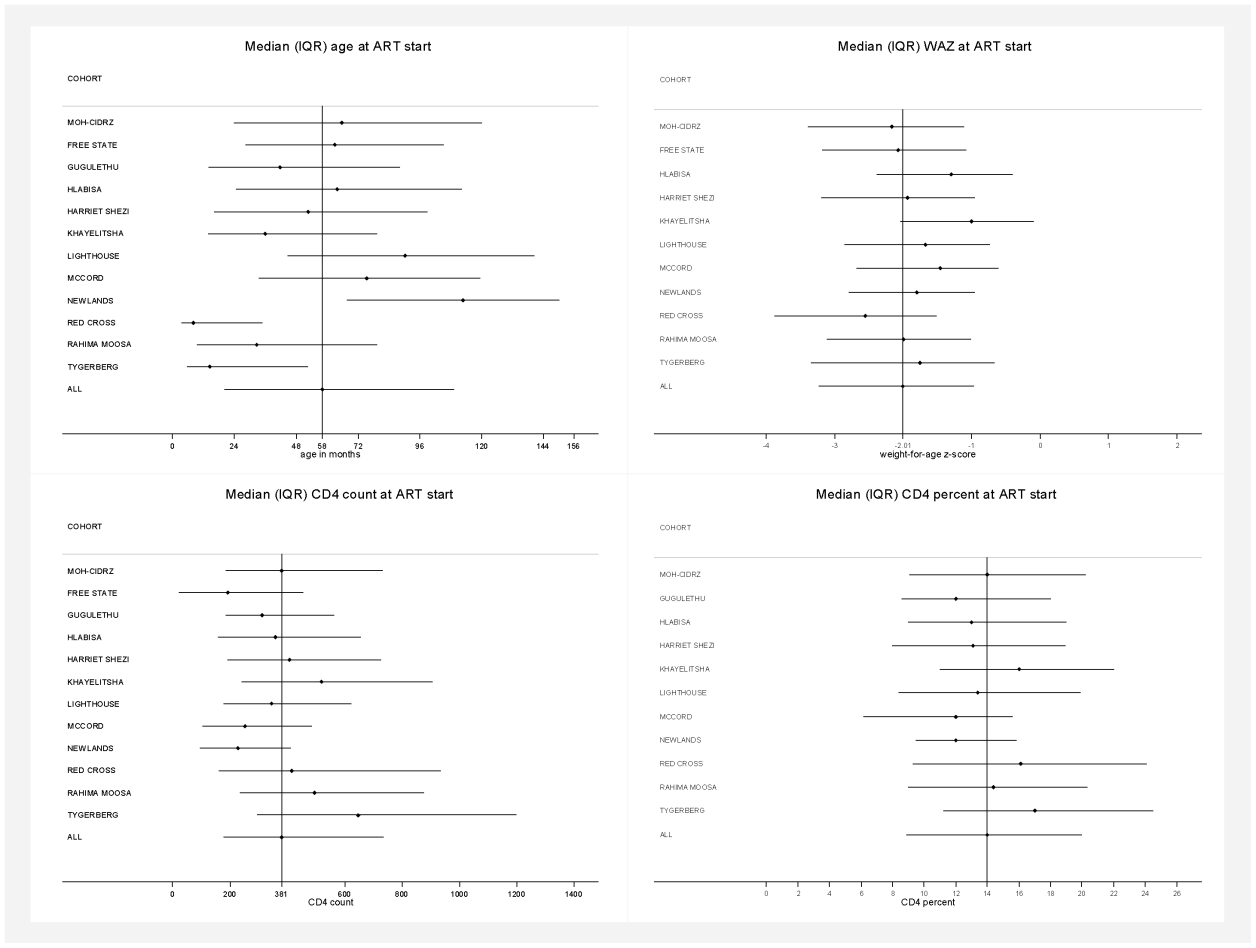
Characteristics of children at ART start in the different cohorts. Characteristics of children at ART start in the different cohorts (median [IQR] age, weight-for-age z-score [WAZ], CD4 count and percent).

Between 2005 and 2010, 30,300 ART-naïve children initiated ART at 12 IeDEA-SA treatment programs in 4 countries and were included in the analysis. There was considerable heterogeneity in program characteristics and the number (median 1,178; range across sites: 402–12,378), and characteristics of children initiating treatment at each program (Table 1 ; Figure 1 ; Supplementary Table S1 in File S1). Programs ranged from single hospital facilities providing pediatric tertiary care to a number of clinics and hospitals providing all levels of care for adults and children across a health district or province. Median (IQR) age at ART initiation was 58 (20–109) months; range across sites 8–113. As expected with such heterogeneity in age, there was variability in CD4 count with median (IQR) of 381 (180–734) cells/mm^3^; range across sites: 193–647. CD4% was less variable with median (IQR) of 14.0% (8.9–20.0); range across sites: 12.0–17.0. Overall, 70% of children were severely immunosuppressed and 72% had WHO Stage III/IV disease with ranges across sites of 54–84% and 28–93% respectively. Median (IQR) WAZ was −2.01 (−3.23 to −0.97) and 29% of children <10 years old had WAZ<−3 (range across sites 13–40%). Variables were frequently not available for all children. Overall the proportions of missing data for WHO Stage, CD4%/count, haemoglobin and weight were 19%, 34%, 47% and 22%. The median (IQR) number of days between variable measurement and ART start date was −21 (−44 to −9) (CD4 count), −23 (−46 to −12) (CD4%), −20 (−45 to 0) (HIV-RNA) −16 (−34 to −6) (hemoglobin) and 0 (0 to 0) for WAZ.

**Table 1:** Characteristics of cohorts.

Cohort name and location	Main level of care provided	Multiple facilities	Type of clinic and payment	Target population	Number of children ever started on ART since program start	Number of children included in the analysis (started ART between 1 Jan 2005 and 31 Dec 2010)
**Zambian MOH-CIDRZ, Lusaka, Zambia**	Primary	Yes	Public, Free ART	Adults and children, combined clinics	14,500	12,378
**Free State Province, South Africa**	All levels	Yes	Public and research, Free ART	Adults and children, combined clinics	4,629	4,579
**Gugulethu Community Health Centre, Cape Town, South Africa**	Primary	No	Public and research, Free ART	Adults and children, separate clinics	483	402
**Hlabisa HIV Care and Treatment Programme, Kwazulu-Natal, South Africa**	Primary	Yes	Public, Free ART	Adults and children, combined clinics	1141	1134
**Harriet Shezi, Chris Hani Baragwanath Hospital, Soweto, South Africa**	All levels	No	Public and research, Free ART	Children only	4,629	3,996
**Khayelitsha Community Health Centre, Cape Town, South Africa**	Primary	Yes	Public, Free ART	Adults and children, separate clinics	1,191	919
**Lighthouse Clinic, Kamuzu Central Hospital, Lilongwe, Malawi**	All levels	No	Public, Free ART	Adults and children, separate clinics	1,416	1,221
**McCord Hospital, Durban, South Africa**	Secondary	No	Private not-for-profit; Small co-payment	Adults and children, separate clinics	804	735
**Newlands Clinic, Harare, Zimbabwe**	Secondary	No	Public, Free ART	Adults and children, separate clinics	874	829
**Red Cross Children's Hospital, Cape Town, South Africa**	Tertiary	No	Public and research; Free ART	Children only	1,873	1,360
**Rahima Moosa Mother and Child Hospital, Johannesburg, South Africa**	All levels	No	Public, Free ART	Children and pregnant women	2,198	1,854
**Tygerberg Hospital, Cape Town, South Africa**	Tertiary	No	Public and research, Free ART	Adults and children, separate clinics	1,307	893
**TOTAL**					**35,045**	**30,300**

### Changes in guidelines and temporal trends in child characteristics

**Figure 2:**
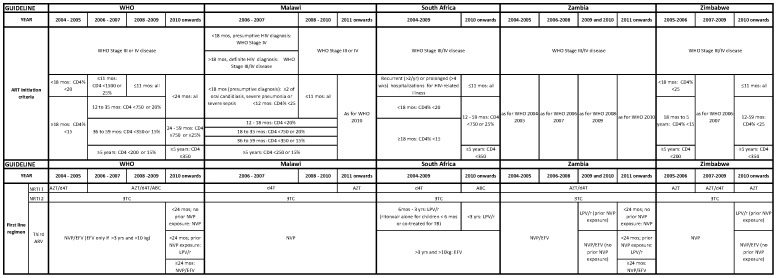
WHO and country antiretroviral therapy guidelines for children from 2004–2011.

Most programs followed national guidelines for ART initiation and first-line regimen choice which were based on WHO guidelines (Figure 2) [24], [25], [26], [27], [28], [29]. There were sometimes lags or minor discrepancies between WHO recommendations and national guidelines. For example, while the WHO 2010 guidelines recommend ART initiation for all children <24 months old irrespective of disease severity, this was implemented in Zambia in 2011. South Africa implemented ART irrespective of disease severity for infants <12 months in April 2010, but this was only expanded to children 12–24 months of age in 2012, when ART for all children <5 year of age irrespective of disease severity was recommended [30].

**Figure 3:**
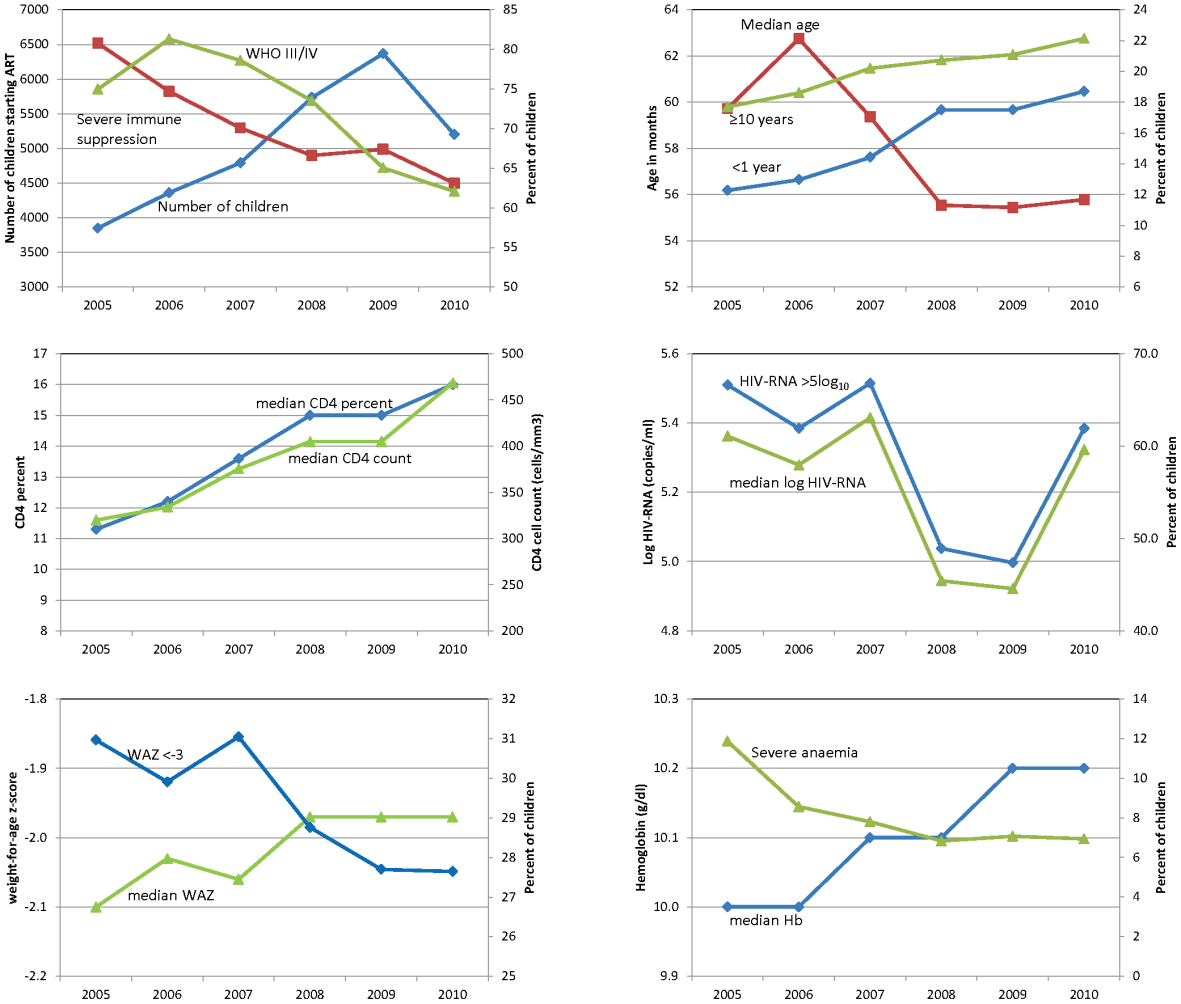
Characteristics of children at ART start by program year. Characteristics of children at ART start by program year summarized as medians for continuous variables and proportions for categorical variables. p<0.001 for changes over program year for all variables (Cuzick's test for trend for continuous variables and logistic regression for categorical variables). (Note: Only children <10 years of age included in weight-for-age z-score graphs and only South African sites included for graphs of HIV-RNA.)

The number of children starting therapy increased each year from 3,849 in 2005 to 6,368 in 2009 (Figure 3). There was a slight decline in 2010, however 2 programs transferred data before the year ended. The median age dropped from a peak of 63 months in 2006 to 56 months in 2010 (p<0.001). Both the proportion of children <1 year and ≥10 years increased over time from 12 to 19% (p<0.001) and 18 to 22% (p<0.001) respectively. Children had less severe disease at ART initiation in later years for almost all markers – baseline CD4%/count increased and there were significant declines in the proportions with severe immunosuppression (81 to 63%; p<0.001), WHO Stage III/IV disease (75 to 62%; p<0.001) and severe anemia (12 to 7%; p<0.001) (Figure 3 ; Supplementary Table S2 in File S1). Nutritional status improved slightly e.g. the proportion with WAZ<−3 decreased from 31 to 28% (p<0.001) and other nutritional indices showed similar trends. Completeness of data for most variables increased over time e.g. the proportion with missing data on weight and hemoglobin dropped from 30 to 18% and 60 to 41%. Notable exceptions were CD4%/count where the proportion of missing data was constant and HIV-RNA where it increased from 43% to 61%. Median HIV-RNA and proportion with >5 log_10_ copies/ml showed a curious pattern of first decreasing and then increasing in 2010, however there was still a significant trend to lower values in the later years.

Temporal trends were mostly similar when looking separately at sites from within and outside South Africa (Supplementary Table S3 in File S1). The only notable difference was in the proportion of children in different age groups. The proportion of children <1 year of age increased in later years across the entire region, but the proportion in South Africa in 2010 (25.7%) was much higher than in the rest of the region (12.7%). The proportion of children aged 1–2 years increased in sites from outside South Africa, but not in South African sites. The proportion of children ≥10 years increased in South Africa but decreased in sites from outside South Africa.

### Temporal trends in characteristics of children <1 year of age

**Figure 4:**
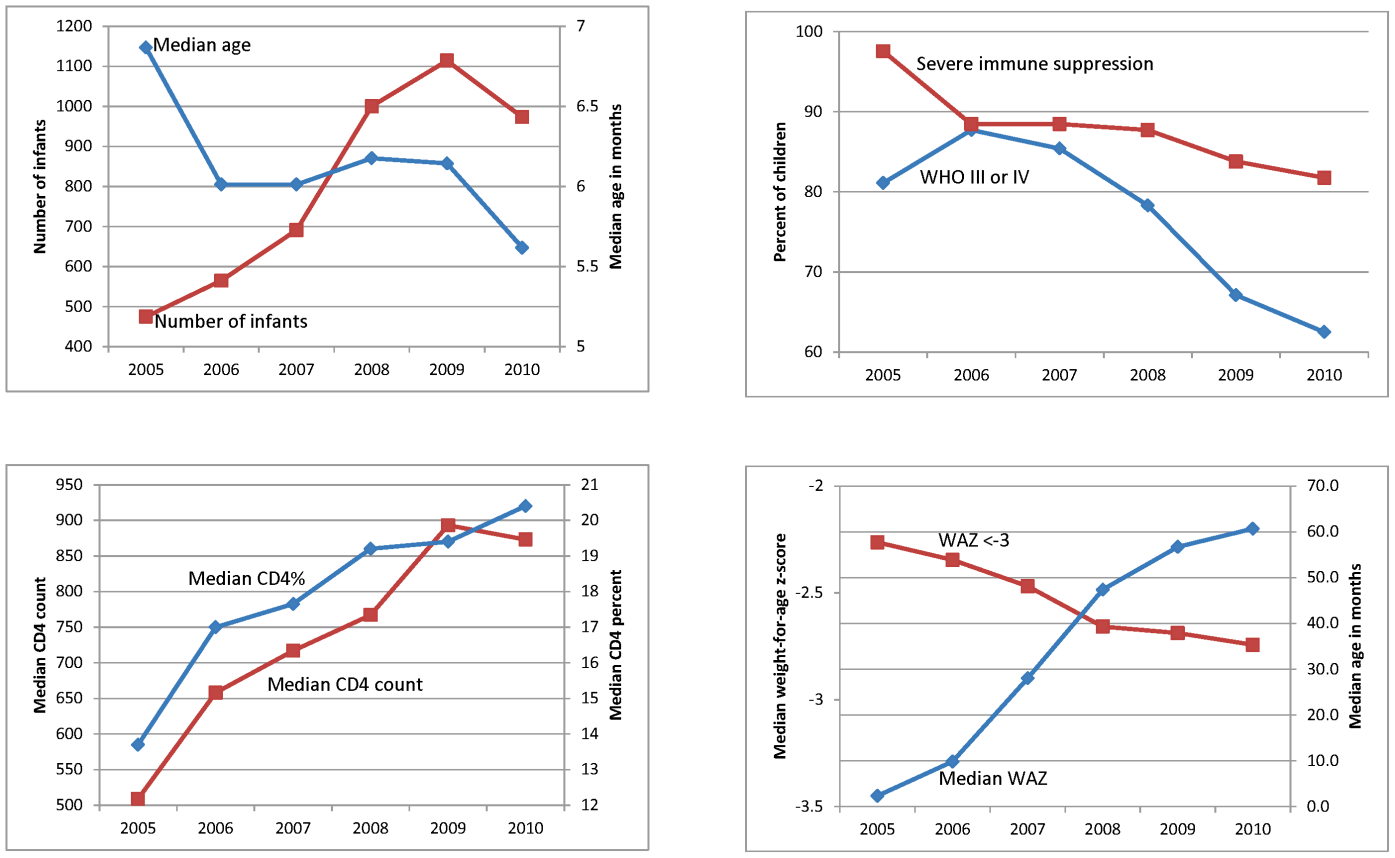
Characteristics of children <1 year of age at ART start by program year. Characteristics of children <1 year of age at ART start by program year summarized as medians for continuous variables and proportions for categorical variables. p<0.001 for changes over program year for all variables (Cuzick's test for trend for continuous variables and logistic regression for categorical variables).

The number of children <1 year old at ART initiation more than doubled from 475 in 2005 to a peak of 1,114 in 2009, with a slight drop in 2010 (Figure 4 , Supplementary Table S4 in File S1). Median age at ART start in children <1 year dropped from 6.9 to 5.6 months. Children <1 year at ART start in later years had less severe disease. Median CD4%/count increased (p<0.001) and the proportion with severe immunodeficiency dropped from 98 to 82% and with WHO Stage III/IV disease from 81 to 63%. In 2005 nearly 60% of infants were severely underweight at ART start and this declined to 35% in 2010, with similar improvements in HAZ and BAZ.

### First-line regimen

Almost all first-line regimens were an option selected from nucleoside/nucleotide reverse transcriptase inhibitor (NRTI)1 (stavudine [d4T]/zidovudine [AZT]/abacavir [ABC]/tenofovir [TDF])+NRTI2 (lamivudine [3TC]/emtricitabine [FTC])+protease inhibitor (PI)/non-nucleoside reverse transcriptase inhibitor (NNRTI). First-line regimen differed for sites from within and outside South Africa and for children younger and older than 3 years. This is in keeping with national guidelines and drug prescribing information – dosing for efavirenz (EFV) was not established for children <3years/10 kg; outside South Africa nevirapine (NVP) was recommended as the “third drug” for all children, while South African guidelines recommended PIs for children <3 years/10 kg, and EFV in older children. Across all age groups, 3TC predominated as NRTI2.

*NRTI1*: Outside South Africa d4T use was predominant and increased over time from 61% to 81% (p<0.001) and 66% to 71% (p<0.001) in children below and above 3 years of age, with corresponding declines in AZT use. There were also small increases over time in ABC use for all children and TDF for children >3 years. Within South Africa d4T use increased slightly in children <3years from 2005 to 2009 (83 to 89%) with corresponding declines in AZT, but was constant at 96–97% in older children. In both age groups a change was seen in 2010 with proportion starting d4T dropping to 41% and 60% in children younger and older than 3 years, with ABC replacing d4T as the NRTI1 of choice. There was negligible use of TDF.

*NNRTI/PI*: Outside South Africa NVP use was predominant in both age groups: 97 and 87% of children younger and older than 3 years. Almost all of the remainder of children initiated EFV, use of which increased over time in older children from 8 to 16% (p<0.001). There was negligible use of PIs. Inside South Africa in children <3 years, NVP use declined from 9% in 2005 to <1% in 2010 (p<0.001) with corresponding increases in lopinavir-ritonavir (LPV/r) use. In 2005 and 2006, 11% of children <3 years initiated therapy with ritonavir alone as the third drug, but this was almost completely eliminated from 2007 onwards when LPV/r dosing recommendations for children <6 months of age were introduced and double dose LPV/r or ritonavir-boosted LPV/r were used when co-treating for tuberculosis. EFV predominated in children >3 years (93% of children) with no temporal changes.

## Discussion

This analysis of >30,000 children who started ART across Southern Africa between 2005 and 2010 demonstrates that not only is the number of children starting therapy increasing, but also that in recent years children are younger and less severely ill at ART start. Nevertheless, even in 2010 many children were still initiating therapy with advanced disease −62% with WHO Stage III/IV disease, 29% with WAZ<−3 and 63% were severely immunosuppressed.

Similar temporal trends in disease severity characteristics were noted in a South African study of 3,000 children initiating therapy at 30 health facilities between 2005 to 2009 [13], as well as in Zambia [14], Both the South African and Zambian studies showed temporal improvements in mortality [13], [14]. Initiating therapy earlier in the course of disease is important as children with less severe disease experience better outcomes on ART and, in particular, the high early mortality seen in children starting therapy in resource-limited settings may be attenuated [7], [31], [32], [33], [34], [35], [36]. Early ART also improves neurodevelopmental outcomes in infants [12]. As has been found in adult studies, however, in Zambia there was also increasing loss to follow-up over time which may include unascertained mortality [14], [37], [38]. Encouragingly in the South African study loss to follow-up did not increase over time [13].

It appears there is a mixed pattern of temporal trends in age with increasing proportions of infants as well as older children and adolescents initiating therapy, although the latter was only seen at South African sites [13]. Despite temporal increases, the proportion of infants initiating therapy in our study was still low especially in sites outside South Africa, reaching 19% across the region in 2010. Nevertheless this is substantially higher than in previously published studies and may be due to the inclusion of data from 2010 after most countries had adopted universal ART for children <1 year, as well as inclusion of 2 sites providing exclusive tertiary care in South Africa where >50% of children were infants. It is notable that there was no increase in the proportion of children aged 1–2 years at ART initiation in South Africa, as this is the only country in the region where the WHO 2010 recommendation of ART initiation irrespective of disease severity was restricted to children <1 year, rather than <2 years of age as was adopted in other countries. The proportion of children starting therapy in infancy is complex as it is a composite result of the ability of health care services to promptly diagnose and initiate infants on therapy, the coverage and effectiveness of PMTCT programs in reducing numbers of newly infected infants, as well as the backlog of older children not yet initiated on therapy. Indeed, the curious increase in median log_10_ viral load values in 2010 may be due to both the younger age of infants starting ART and increasing coverage and effectiveness of PMTCT, with high HIV-RNA values in young infants infected despite PMTCT exposure [30]. The backlog of older children not yet on ART may be substantial and is an important consideration in designing pediatric HIV care and ART programs as the needs and disease spectrum in these children are different from infants and toddlers [39], [40], [41]. In our study 1,151 children ≥10 years of age (22% of all children) initiated therapy in 2010, however these were not all confirmed to be infected perinatally, rather than through sexual transmission. Other studies have found considerable numbers of perinatally infected children being diagnosed with HIV in late childhood or adolescence [39], [42], [43].

Despite temporal improvements across all ages in disease severity at ART start, the proportion of children initiating therapy with severe disease even at the end of the analysis period is a concern. Even in 2010, the median CD4%, WAZ and log_10_ HIV-RNA in our study indicated markedly more severe disease than those from a review of studies from developed countries [8]. This highlights the need to scale up provider-initiated testing and counselling for children at all health care encounters such as vaccination clinics, outpatient and inpatient services in order to ensure prompt diagnosis of HIV and initiation of therapy in those that need it [44], [45]. In this respect the recently updated WHO 2013 guidelines [46] recommending ART initiation in all children <5 years of age irrespective of disease severity may reduce barriers to starting ART and streamline initiation. Delayed ART initiation is a particular concern for infants where early ART before disease progression substantially reduces mortality and morbidity [18]. In our study in 2010, >60% of infants started therapy with WHO Stage III/IV disease and >80% with severe immune suppression. The median age of infants starting ART in 2010 was 5.6 months which is well above the median age of <2 months in the immediate arm of the CHER Study. There are a number of barriers to early ART initiation including lack of access to HIV-DNA PCR testing for diagnosis [24], [47], [48], [49], poor integration of antenatal, PMTCT, maternal and child health (MCH) and HIV services with poor infant HIV testing even among those whose mothers enrolled in PMTCT care [50], lack of expertise, experience and confidence with initiating and maintaining infants on ART and poor availability of drugs in suitable formulations for infants [4], [51]. Strategies to improve ART access for infants such as integration of PMTCT, MCH and HIV services and provider initiated testing at vaccination and other health visits [45], [52], [53], [54] need to be developed and expanded. In Zambia, the median age of children enrolling in HIV care declined in settings where there was close collaboration between the MCH service and the ART clinic [14]. Notwithstanding these measures, disease progression in infants, especially those infected *in utero*, is extremely rapid −25% of 560 HIV-infected infants identified at <12 weeks old as potential participants in the CHER study were ineligible as they already had CD4%<25, symptomatic disease or had died before screening/enrolment [18]. Currently infant diagnostic testing is recommended at 6 weeks of age, with ART initiation in infected infants at 10–14 weeks of age. However, in a study to determine optimal timing of infant diagnostic testing, it was found that 45% of *in utero*-infected and 22% of *intrapartum*-infected infants had died or were lost to follow-up by 14 weeks of age [55], [56]. The role of earlier PCR testing in infants needs to be explored.

First-line regimen use closely followed national guidelines. PI use was almost completely absent outside of South Africa, but this is likely to change. The WHO 2013 Guidelines [46] now recommend PI-based regimens for all children <3 years old. The P1060 trial demonstrated better survival and virological outcomes in children <3 years old initiating PI based compared to NNRTI-based therapy, irrespective of prior PMTCT exposure [57], and a resistance surveillance study in Zimbabwe found substantial NNRTI resistance in children <18 months old without prior exposure to NVP [58]. Similarly while TDF use was very limited, this is likely to increase among older children. The feasibility, cost, safety and long term outcomes of these changes will need to be examined.

To our knowledge this is the largest study of temporal trends in child characteristics at the start of ART in sub-Saharan Africa and the only study including a range of settings in more than one country to date. The number of children in our study is nearly 10% of the 337,200 children estimated to be receiving ART in eastern and southern Africa by the end of 2010 [1]. Inclusion of data from 3 different periods of WHO guidelines enabled us to comment on how patient characteristics at ART start have changed as a result of WHO and national guideline changes. Representativeness of all children initiating ART in the region may however be limited as cohorts contributing to IeDEA-SA must have electronic patient databases and so are likely to be better resourced than all programs in the region. Nevertheless, the use of routine program data from busy clinics in this analysis, rather than dedicated research databases, enhances representativeness. There was substantial missing data on a number of variables, but this is true of routine monitoring data from many settings, and it is encouraging that the proportion of missing data for most variables decreased in later years [13], [34]. Notwithstanding, it is a concern that WHO Stage and/or CD4 measurement data is missing for many children as failure to routinely perform these assessments in all HIV-infected children may result in missed opportunities to initiate ART before the onset of advanced disease. It is therefore important to ensure improved access to CD4 testing and results as well as mentorship of health care workers to ensure regular and accurate assessment for new clinical events. The recent WHO recommendation [46] to start treatment in all children <5 years of age irrespective of disease severity should reduce missed opportunities for ART initiation due to lack of Stage or CD4 measurement in these children. Data on key variables of interest such as PMTCT exposure and tuberculosis infection at ART start were too sparse to include in the analysis.

In conclusion, over time increasing numbers of children are initiating ART at younger ages and with less severe disease in these programs. However the proportion of infants remains low and the proportion of children across all ages initiating ART with severe disease remains high. It is important to continue to develop strategies for earlier diagnosis and treatment of infants as well as to examine long term outcomes in the era of expanding coverage of pediatric HIV and more effective PMTCT.

## Supporting Information

File S1 **contains the following:** **Table S1. Characteristics of children at ART initiation in each cohort for (a) continuous variables and (b) categorical variables.** **Table S2. (a) Median (IQR) values of characteristics at antiretroviral therapy initiation for children by program year from 2005 to 2010.** (b) Proportion of children in each age group and with particular disease severity characteristics or missing data by program year. **Table S3. (a) Median (IQR) values of characteristics at antiretroviral therapy initiation for children by program year from 2005 to 2010 shown separately for sites outside South Africa and South African sites.** (b) Proportion of children in each age group and with particular disease severity characteristics or missing data by program year shown separately for sites outside South Africa and South African sites. **Table S4. (a) Median (IQR) values of characteristics at ART initiation for children <1 year of age at ART start by program year from 2005 to 2010.** (b): Proportion of children <1 year of age at ART start with particular disease severity characteristics or missing data by program year from 2005 to 2010.
(DOCX)
