# Supplementary material for: Temporal Trends in the Characteristics of Children at Antiretroviral Therapy Initiation in Southern Africa: The IeDEA-SA Collaboration
Source: PLoS One. 2013 Dec 9;8(12):e81037. doi: 10.1371/journal.pone.0081037 (PMC3867284; doi:10.1371/journal.pone.0081037)
Supplement: File S1 — contains the following: Table S1. Characteristics of children at ART initiation in each cohort for (a) continuous variables and (b) categorical variables. Table S2. (a) Median (IQR) values of characteristics at antiretroviral therapy initiation for children by program year from 2005 to 2010. (b) Proportion of children in each age group and with particular disease severity characteristics or missing data by program year. Table S3. (a) Median (IQR) values of characteristics at antiretroviral therapy initiation for children by program year from 2005 to 2010 shown separately for sites outside South Africa and South African sites. (b) Proportion of children in each age group and with particular disease severity characteristics or missing data by program year shown separately for sites outside South Africa and South African sites. Table S4. (a) Median (IQR) values of characteristics at ART initiation for children <1 year of age at ART start by program year from 2005 to 2010. (b): Proportion of children <1 year of age at ART start with particular disease severity characteristics or missing data by program year from 2005 to 2010. (DOCX) [file pone.0081037.s001.docx]

**Supplementary Tables**

Supplementary Table S1: Characteristics of children at ART initiation in each cohort for (a) continuous variables and (b) categorical variables

a)

*Children < 10 years of age

^$^South African sites only

b)

NR = Not recorded

^*^South African sites only

^#^Children < 10 years of age

Supplementary Table S2 (a): Median (IQR) values of characteristics at antiretroviral therapy initiation for children by program year from 2005 to 2010.

^*^South African sites only

^#^Cuzick's test for trend over ordered groups

Supplementary Table S2(b) Proportion of children in each age group and with particular disease severity characteristics or missing data by program year.

NR = not reported

^*^South African sites only

^#^p-values derived from logistic regression of binary dependent variable with programme year as an independent variable

Supplementary Table S3 (a): Median (IQR) values of characteristics at antiretroviral therapy initiation for children by program year from 2005 to 2010 shown separately for sites outside South Africa and South African sites.

^*^Cuzick's test for trend over ordered groups

Supplementary Table S3(b) Proportion of children in each age group and with particular disease severity characteristics or missing data by program year shown separately for sites outside South Africa and South African sites.

NR = not reported

^*^p-values derived from logistic regression of binary dependent variable with program year as an independent variable

Supplementary Table S4 (a): Median (IQR) values of characteristics at ART initiation for children <1 year of age at ART start by program year from 2005 to 2010.

^*^South African sites only

^#^Cuzick's test for trend

Supplementary Table S4 (b): Proportion of children <1 year of age at ART start with particular disease severity characteristics or missing data by program year from 2005 to 2010.

^*^South African sites only

NR = not recorded

^#^p-values derived from logistic regression of binary dependent variable with programme year as an independent variable
